# Antimicrobial Activity of Small Synthetic Peptides Based on the Marine Peptide Turgencin A: Prediction of Antimicrobial Peptide Sequences in a Natural Peptide and Strategy for Optimization of Potency

**DOI:** 10.3390/ijms21155460

**Published:** 2020-07-30

**Authors:** Ida K. Ø. Hansen, Tomas Lövdahl, Danijela Simonovic, Kine Ø. Hansen, Aaron J. C. Andersen, Hege Devold, Céline S. M. Richard, Jeanette H. Andersen, Morten B. Strøm, Tor Haug

**Affiliations:** 1 Norwegian College of Fishery Science, Faculty of Biosciences, Fisheries and Economics, UiT The Arctic University of Norway, 9037 Tromsø, Norway; ajca@dtu.dk (A.J.C.A.); hege.devold@uit.no (H.D.); celine.s.richard@uit.no (C.S.M.R.); 2 Department of Pharmacy, Faculty of Health Sciences, UiT The Arctic University of Norway, 9037 Tromsø, Norway; tlovdahl88@hotmail.com (T.L.); danijela.simonovic@uit.no (D.S.); morten.strom@uit.no (M.B.S.); 3 Marbio, Faculty of Biosciences, Fisheries and Economics, UiT The Arctic University of Norway, Breivika, N-9037 Tromsø, Norway; kine.o.hanssen@uit.no (K.Ø.H.); jeanette.h.andersen@uit.no (J.H.A.)

**Keywords:** Arctic, ascidian, antimicrobial, synthetic, peptide, *Synoicum turgens*

## Abstract

Turgencin A, a potent antimicrobial peptide isolated from the Arctic sea squirt *Synoicum turgens*, consists of 36 amino acid residues and three disulfide bridges, making it challenging to synthesize. The aim of the present study was to develop a truncated peptide with an antimicrobial drug lead potential based on turgencin A. The experiments consisted of: (1) sequence analysis and prediction of antimicrobial potential of truncated 10-mer sequences; (2) synthesis and antimicrobial screening of a lead peptide devoid of the cysteine residues; (3) optimization of in vitro antimicrobial activity of the lead peptide using an amino acid replacement strategy; and (4) screening the synthesized peptides for cytotoxic activities. In silico analysis of turgencin A using various prediction software indicated an internal, cationic 10-mer sequence to be putatively antimicrobial. The synthesized truncated lead peptide displayed weak antimicrobial activity. However, by following a systematic amino acid replacement strategy, a modified peptide was developed that retained the potency of the original peptide. The optimized peptide **StAMP-9** displayed bactericidal activity, with minimal inhibitory concentrations of 7.8 µg/mL against *Staphylococcus aureus* and 3.9 µg/mL against *Escherichia coli*, and no cytotoxic effects against mammalian cells. Preliminary experiments indicate the bacterial membranes as immediate and primary targets.

## 1. Introduction

Antibiotic-resistant bacteria are emerging as a major global health problem and are considered one of the biggest future medical threats to humankind. Many pathogenic bacteria previously susceptible to antibiotics are now becoming nearly impossible to combat [1,2,3]. The increasing number of immunocompromised patients (AIDS, cancer and transplant recipient patients) and the rising number of elderly further aggravate the problem, as they often need effective antibiotics to treat infections caused by opportunistic bacteria [4,5,6]. Currently, infections caused by antibiotic-resistant bacteria are estimated to cause more than 700,000 deaths annually and the number is rising [7]. Due to a long-term focus on the modification of existing conventional antibiotics by the pharmaceutical industry, rather than development of novel treatment options, modern medicine is now in dire need of a solution to the problem [8]. Antimicrobial peptides have, in the last three decades, gained increasing attention as promising candidates to solve the challenges of antibiotic resistance [9].

Natural AMPs usually consist of less than 60 amino acid residues, which occur mainly in the natural L-configuration, and have molecular masses below 10 kDa [10,11]. AMPs normally have a substantial portion of hydrophobic residues (≥30%) and most are cationic, with a net charge of +2 to +9 [12]. The segregated arrangement of the hydrophobic and cationic amino acids gives AMPs an amphipathic nature, a feature allowing interaction with and embedding into anionic microbial cell membranes, causing bacterial death [13]. Thus, the presence of positive charges (mainly caused by the cationic amino acids Lys and Arg) in combination with hydrophobic residues have a fundamental role in the mechanism of action of these potent compounds [14]. Compared to conventional antibiotics, AMPs are substantially less prone to resistance development due to their mode of action, and they exert their killing activity faster (within seconds to minutes) [15]. AMPs often show a wide range of antimicrobial bioactivities, acting as antibacterial, antifungal and antiparasitic agents, and are often highly membrane-selective [11]. Furthermore, linear AMPs can easily be synthesized due to their relatively small size [16]. In this regard, the possibility for AMPs to overtake the title of next-generation antibiotics looks realistic [17]. However, to date, no AMP has reached the antibiotic market, although many AMPs are in clinical trials [18]. The biggest challenges faced in the development of AMPs into drugs are high production costs (especially for large and disulfide-rich peptides), lack of proteolytic stability, and unfavorable toxicology profile when administered systemically [3,17]. To overcome these issues, the pharmacophore of AMPs and the structural features causing toxicity must be identified to enable the production of peptides with improved therapeutic indexes. Furthermore, pharmacophore identification will lower production costs, as only substructures of the peptides need to be produced. This knowledge can be acquired through synthesis of analogues followed by bioactivity testing and structure–activity relationship studies. In fact, recent studies have shown that potent, short (<15 amino acids), linear AMPs (devoid of cysteines), can be successfully produced [3,19]. Certain characteristics have also proved to play a critical role for the activity of these peptides, like the balance between the positive charge, hydrophobicity, and content of lipophilic bulky residues such as Trp [19]. These peptides have shown effectiveness against bacterial infections in vivo [20], as well as improved stability in serum [21]. By experience, shortened peptides derived from natural AMPs can retain relevant biological activities [22,23]. Consequently, they are excellent candidates as lead peptides for developing novel antimicrobial drugs [2].

Recently, we have characterized two novel AMPs, turgencin A and turgencin B, from the Arctic sea squirt *Synoicum turgens* (Phipps, 1774) [24]. The turgencins are composed of 35–36 amino acid residues with six Cys residues engaged in three disulfide bridges with connectivity of Cys1-Cys6, Cys2-Cys5, and Cys3-Cys4, making them challenging to synthesize and explore as drug leads. The aim of the present study was to make a truncated AMP derivative based on turgencin A with drug lead potential. We recognized an internal stretch (residues 18–27) of turgencin A having an unusual amino acid PGGW central core, flanked by two lysine residues on each end, making it highly cationic. We therefore hypothesized that this 10-residue-long sequence could be used for the generation of a novel antimicrobial lead peptide. The antimicrobial potential of this first lead peptide **StAMP-1** was verified using publicly accessible and pre-trained AMP prediction tools that rely on various machine learning algorithms [25,26] before being synthesized and tested. First, a single-cysteine residue within the sequence was replaced by alanine to avoid potential and unpredictable dimerization. Subsequently, an amino acid replacement strategy was chosen to improve the antimicrobial activity of **StAMP-1**. This involved enrichment of the central core (PGGW) of the peptide with Trp residues, causing an increase in the hydrophobic ratio while leaving the cationic residues unchanged (i.e., ensure a high net positive charge). After optimizing hydrophobicity and exploring sequence effects through the preparation of peptides **StAMP-2-8**, an increase in antimicrobial potency was further explored by synthesizing two peptides, **StAMP-9** and **StAMP-10**, where all four Lys residues were substituted by Arg. Finally, the effects on the antimicrobial activity of Leu and Trp as lipophilic residues was compared by synthesis of **StAMP-11**.

## 2. Results and Discussion

### 2.1. Sequence Analysis and AMP Prediction

**Figure 1:**
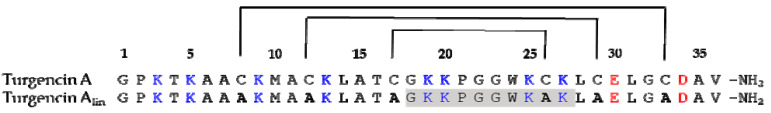
Amino acid sequences and disulfide bond connectivity of turgencin A and its linear derivative turgencin A_lin_ where all Cys residues were replaced by Ala (shown in bold). The potential 10-residue lead peptide sequence containing the PGGW core is shaded in grey.

Turgencin A is a potent AMP consisting of 36 amino acid residues with six cysteines forming three intramolecular disulfide bridges. Sequence homology with turgencin B indicates that these bridges are formed between Cys8-Cys33, Cys12-Cys29 and Cys17-Cys26 (Figure 1) [24]. By experience, short peptide segments derived from larger natural AMPs can partly be responsible for the detected activity [22,23,27], and thus be promising lead sequences for drug development. By visual inspection and similarity searches in various AMP databases (for review, see Liu et al. [28]), we recognized a cationic region within turgencin A (sequence 18–27, GKKPGGWKCK) with a 4-amino acid central core sequence PGGW (Figure 1) which was found in some abaecins, a well-known family of AMPs found in insects [29]. Hydrophobic Trp residues are widely accepted as contributors to the bioactivity of AMPs [30,31], and both Pro and Gly residues are known to break up α-helical sequences [32]. Turgencin A also contains an N-terminal Gly residue, which is found to be beneficial in many AMPs [33,34]. We therefore hypothesized that this 10-residue sequence could be used for the generation of novel antimicrobial lead peptides. To support our hypothesis, a linear version of turgencin A (denoted turgencin A_lin_), where all Cys were replaced by Ala, was examined using the online prediction tool of the collection of antimicrobial peptides (CAMP_R3_) web server (Figure 1). CAMP_R3_ contains information on conserved AMP sequence signatures captured as patterns and Hidden Markov Models (HMMs), and currently the database contains 10,247 sequences and 114 family-specific signatures of AMPs [25].

The antimicrobial potential of shortened, overlapping peptides (10-mers), using a sliding window strategy, was predicted by utilizing all four available prediction models: support vector machine (SVM), random forests (RF), artificial neural network (ANN), and discriminant analysis (DA). SVM, RF and DA predict and state a peptide’s probability of having antimicrobial properties in values between 0 (low probability) and 1 (high probability), with values above 0.5 defined as being most likely to be bioactive, whereas the ANN model makes a qualitative statement of either AMP or non-AMP (NAMP). The antimicrobial potential of these peptides was also predicted using a different SVM model available through ADAM, another comprehensive AMP database, containing 7007 unique sequences. In this model, a higher value indicates higher probability for antimicrobial activity. [26]. Out of the 27 sequences analyzed, only one sequence (sequence 18–27, GKKPGGWKAK) was predicted to be antimicrobial by all four models in AMP_R3_, including the RF classifier, and by the SVM model in ADAM (Table 1). Although several other peptides were predicted to be antimicrobial by the SVM, DA and ANN models, only one additional sequence (the neighboring sequence, 19–28) was predicted to be active by the RF model (RF value > 0.5). A number of AMP prediction tools have been designed attempting to discriminate AMPs from non-AMPs (NANP) (reviewed by Liu et al. [28]). Among ten web-based AMP prediction tools, the CAMP_R3_ (RF) tool was recently shown to outperform other web-based prediction models, followed by CAMP_R3_ (SVM) and ADAM (SVM) [35]. The Lys residues, which are spread through the turgencin A sequence, provide a positive charge to the peptide. Thus characteristic is known to be important for the activity of most AMPs. Of notice, the two 10-mer sequences predicted to be active by the RF model were also the two peptides with the highest net positive charge (+4) (Table 1). Furthermore, the peptide sequence, 18–27 (GKKPGGWKAK), was calculated to have a Boman index of 1.52, the highest among all the predicted peptides, but still in the middle range among AMPs [36]. The Boman Index is an estimate of protein-binding potential, calculated on the basis of cyclohexane-to-water partition coefficient of the respective amino acid side chains divided by the total number of amino acid residues within the peptide [36]. A high index value (>2.48) indicates a multifunctional peptide with high binding potential (e.g., hormones). A low index value (≤1) indicates a potential AMP with less side effects (e.g., low hemolytic activity) [36]. Based on the above predictions, the C-terminally amidated sequence 18–27 (GKKPGGWKAK; hereafter named **StAMP-1**) was selected as the first lead peptide to be synthesized, screened for antimicrobial activity, and further optimized employing an amino acid replacement strategy. Through single-residue substitutions, additional peptides were rationally designed with the purpose of increasing the hydrophobic ratio, i.e., overall hydrophobicity, and to investigate sequence specific effects. This resulted in the production of ten additional truncated turgencin A analogs, **StAMP-2-11**.

### 2.2. Peptide Design and Antibacterial Screening

A high net-positive charge is vital for many cationic AMPs, predominantly with regard to initial electrostatic interaction with the anionic microbial cell surfaces and subsequent disruption of the bacterial cell membrane or intracellular translocation [30,37,38]. The lead peptide **StAMP-1** and all proceeding peptides were therefore synthesized with an amidated C-terminal end, which provides an increase in net positive charge by +1 by masking the otherwise anionic C-terminal carboxylate group. The original turgencin A peptide is also amidated C-terminally [24]. An amidated C-terminus can also provide resistance to the action of carboxypeptidases, as shown for the well-known AMP magainin [39]. An overview of the synthesized **StAMP-1-11** peptides and their physicochemical characteristics is presented in Table 2.

As predicted by four models in the CAMP_R3_ web server, **StAMP-1** displayed antibacterial activity in vitro (Table 3). However, the prediction models do not predict the exact antimicrobial potency of a given peptide sequence, they only predict the probability of being an AMP. As shown in Table 3, **StAMP-1** displayed low antibacterial activity (MIC = 250 µg/mL) and only against two out of seven test strains: the Gram-positive (G+) bacteria *Bacillus megaterium* and *Corynebacterium glutamicum.* The reason for the weak antibacterial activity was suspected to be a too low hydrophobic ratio (20%, Table 2). According to the APD3 database, the average hydrophobic ratio of AMPs deposited in the database is about 41.5% [40]. Higher hydrophobicity would ease the penetration of the peptide into the lipid environment of the microbial membranes. However, the hydrophobicity should not be too high, making the peptide insoluble in aqueous environments. On the other hand, according to a recent study [19], short AMPs have different features compared to larger AMPs. Short AMPs do not seem to need structural requirements like high α-helicity, a specific hydrophobic moment, an explicit partitioning of charge and hydrophobicity, or a high frequency of particular amino acids or amino acid pairs (e.g., Arg-Trp or Arg-Arg pairs) within the peptide sequence. For short cationic AMPs, a balance between positive charge and hydrophobicity seems to be more important, and some, but not too many, Trp residues seem to be advantageous [19]. In silico prediction of antimicrobial activity of very short peptides may, therefore, currently be challenging.

The above-mentioned design of **StAMP-2-11** involved enrichment with one (**StAMP-2/3/4**) or two (**StAMP-5/6/7**) lipophilic tryptophan residues in the central core (PGGW) of the first lead peptide **StAMP-1** while leaving the cationic residues (positive charge) unchanged. The substitution of Pro4, Gly5 or Gly6 with a single Trp (**StAMP-2/3/4**) resulted in increased hydrophobic ratio (30%, Table 2) and increased antibacterial activity against three to four of seven bacterial strains, but still only activity against G+ bacteria (and fungi; discussed below) (Table 3). All three peptides (**StAMP-2/3/4**) were highly potent against *B. megaterium*, and out of these three peptides, **StAMP-4** was the overall most potent peptide, having a MIC value of 3.9 µg/mL against both *B. megaterium* and *C. glutamicum*.

Introducing two Trp residues in the PGGW core (**StAMP-5/6/7**), and thereby increasing the hydrophobicity to 40% (Table 2), resulted in further improved antibacterial activity and measurable activity against Gram-negative (G-) bacteria. **StAMP-7**, having a total of three consecutive Trp residues, was overall the most potent peptide in this series, and the only peptide showing antibacterial activity against all test strains to date. **StAMP-7** displayed MIC values in the range of 1.0–125 µg/mL against G+ strains and MIC values of 31.3–250 µg/mL against G- strains (Table 3). The least sensitive bacterial strains within each class were *Staphylococcus aureus* (G+) and *Pseudomonas aeruginosa* (G-), in which *P. aeruginosa* often is the least susceptible strain to many AMPs [24]. Gly does not have a side chain and therefore provides increased flexibility within the sequence of AMPs. This feature does not seem to be of importance to **StAMP-7**, in which both Gly residues in the PGGW core were replaced by Trp. The substitution of Pro4 with Trp in both **StAMP-5** and **StAMP-6** resulted in a lower increase in antimicrobial activity and may indicate a role of Pro4 in peptide folding. Among the two Gly residues, the replacement of Gly5 with Trp (resulting in **StAMP-5**) seemed to result in an improvement in antibacterial activity compared to replacement of Gly6 with Trp (resulting in **StAMP-6**). In general, the antimicrobial activity increased when the overall hydrophobicity of the peptides increased, as measured by RP-HPLC and as shown by the in silico calculations (Table 2). However, increasing the hydrophobicity further by substituting all amino acids in the central core with Trp, giving **StAMP-8** with four consecutive Trp’s and a WWWW core, did not result in a major increase in antibacterial activity except against *P. aeruginosa* (MIC = 125 µg/mL, Table 3). Other studies have shown that there should be a balance between positive charge, hydrophobicity and the amount of tryptophan in small peptides. Peptides with an imbalance between these properties have proven to give weak activity [19]. A hydrophobicity window for the optimal antibacterial activity of AMPs has also been observed by others [41].

After optimizing hydrophobicity and positioning of the inserted tryptophan residues, an attempt to further increase the antimicrobial potential of **StAMP-7** was performed by synthesizing a derivative where all four Lys residues were substituted by Arg. The results for **StAMP-9** where Lys2, Lys3, Lys8 and Lys10 were replaced by Arg resulted in 4-fold increase in antibacterial activity against *Escherichia coli* and an 8-fold increase against *P. aeruginosa*, as well as a 16-fold increase in activity against *S. aureus*. Overall, the MIC values of the Arg-enriched peptide **StAMP-9** ranged from 1.0 to 31.3 µg/mL against all seven bacterial test strains. An increase in antimicrobial activity when replacing Lys with Arg is reported for other Trp-rich AMPs [14]. The hydrophobicity of **StAMP-9** was further increased by replacing Pro4 with Trp, resulting in **StAMP-10** with a WWWW core and a hydrophobic ratio of 50% (Table 2). As observed above for **StAMP-8**, this did not result in an improvement in antimicrobial activity, but the contrary, except against *P. aeruginosa* where the activity of **StAMP-9** and **StAMP-10** were similar (Table 3). Leu is, in some scales, reported to supersede Trp in hydrophobicity [42,43], and as a final peptide we made the Leu analog of the most potent peptide **StAMP-9,** resulting in the peptide **StAMP-11**. As shown in Table 3, **StAMP-11**, where all Trp residues were replaced by Leu, showed low antimicrobial activity, except against *B. megaterium* (MIC = 7.8 µg/mL). Thus, the putative high hydrophobicity of Leu was not enough to displace the more advantageous bulkiness of Trp with respect to antimicrobial activity.

When inspecting all the prepared 10-mer peptide sequences, it was noteworthy that the most potent antibacterial peptides within each series retained the Pro4 residue, as shown for **StAMP-4**, **StAMP-7**, and **StAMP-9**, and to some extent also **StAMP-11**. Pro is reported to be an α-helix breaker and that may serve a special function in the peptides by forming a hinge between the three first N-terminal GKK-residues and the following Trp enriched core sequence. In the present peptides, this may have been an important structural feature affecting the overall conformation of the Pro4 containing peptides upon interaction with bacterial membranes. In silico prediction of the antimicrobial potential of the designed 10-mer peptides showed that they were all proposed to be active (Table S1). However, whereas **StAMP-9** was the overall most potent peptide in the antimicrobial screening, **StAMP-8** showed the overall highest scores in the prediction. However, as previously mentioned, the prediction models do not predict the antimicrobial potency of a given sequence, only the probability of being antimicrobial.

#### 2.2.1. Bacterial Killing Experiments

**Figure 2:**
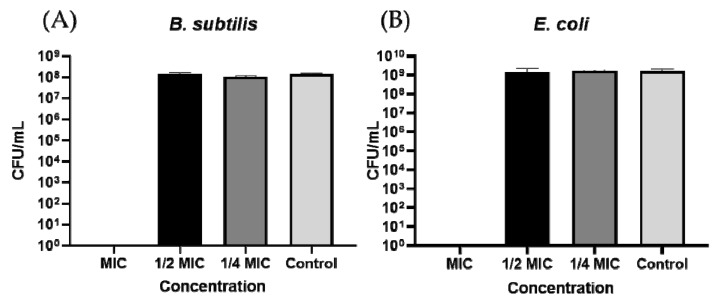
Bactericidal activity of **StAMP-9** against (**A**) *B. subtilis* and (**B**) *E. coli*. Colony-forming units (CFU) per mL were counted after treatment with MIC, ½ MIC, ¼ MIC and no treatment (Control). Each bar presents the mean of three replicates ± SD.

Overall, **StAMP-9** displayed the most potent antibacterial activity of the peptides that were synthesized (Table 3). To evaluate whether the peptide only inhibited growth (bacteriostatic) or killed the bacteria (bactericidal), **StAMP-9** was subjected to a bacterial killing experiment. The G+ bacteria *Bacillus subtilis* and the G- bacteria *E. coli* were selected for the experiment. As illustrated by the bar chart in Figure 2, no colony-forming units (CFU) were formed on the plates treated with overnight cultures that had been incubated with MIC (3.9 µg/mL for *B. subtilis* and 7.8 µg/mL for *E. coli*) or higher concentrations of the peptide. These results suggested that **StAMP-9** was bactericidal at MIC against both bacteria. Lower concentrations of **StAMP-9** produced approximately the same amount of CFU as the control (bacteria and water) after 24 h of incubation.

#### 2.2.2. Membrane Integrity and Viability Investigations

Based on the results from antibacterial screening and the bacterial killing experiment, **StAMP-6-10** were further studied for their immediate effect on the membrane integrity and viability on *B. subtilis* 168 and *E. coli* K12. In the integrity assay, both bacteria are carrying the luciferase *luc*GR gene within the plasmid pCSS962. When a compound disrupts the membrane, externally added D-luciferin can diffuse into the cells and function as a substrate for the luciferase enzyme. This, in turn, causes the emission of light as relative luminescence units (RLU), a signal whose strength is relative to the degree of membrane disruption in living cells. If the test compound affects the membrane sufficiently to cause bacterial death, the RLU signal will increase until the bacterial ATP storage is empty. At this time, the RLU signal will decrease in line with the decreasing ATP concentrations, as the enzymatic reaction gradually stops. D-luciferin does not cross intact membranes at a neutral pH [44]. Following membrane disruption, the RLU will reach its peak and start to decrease due to reduced cell numbers. In the assay setup used herein, RLU was measured over a period of 3 min. The short time period was selected as many membrane disruptive compounds usually affect the bacterial membranes immediately [24,45,46].

**Figure 3:**
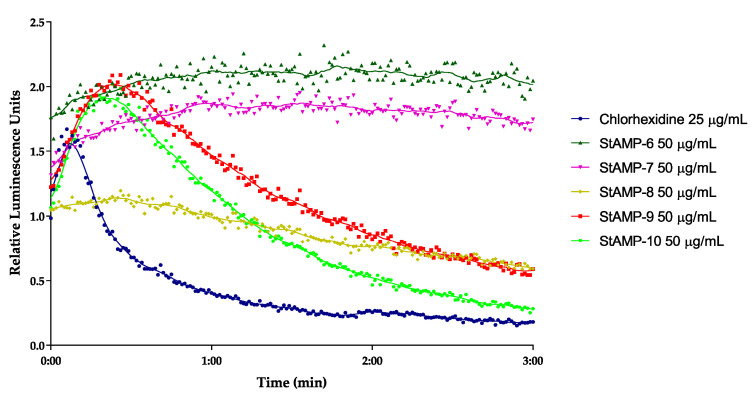
Kinetics of the antimicrobial effect on membrane integrity as measured by relative luminescence emission in *B. subtilis* 168 (pCSS962) in presence of D-luciferin. **StAMP-6-10** and the reference antimicrobial agent chlorhexidine was added to the bacteria. Chlorhexidine served as a positive (membranolytic) control and water as a negative (untreated) control. Each datapoint is the mean of three independent measurements normalized to the negative control.

**StAMP-6-7** affected the membrane integrity of *B. subtilis* at 50 µg/mL (RLU ~ 1.5) (Figure 3). However, since the light emission was not decreasing over time, the membrane disruption was not severe enough that the bacteria were killed within the measured time period. As both peptides gave MIC values of 3.9 µg/mL against the same strain, this might indicate that **StAMP-6-7** had an additional target in *B. subtilis*, or that the membrane disruption process took longer time than 3 min. Concentrations below 50 µg/mL did not affect the membrane.

**StAMP-8-10** showed a stronger disruptive effect on bacterial membrane integrity of *B. subtilis* at 50 µg/mL. As shown in Figure 3, an increase in light emission was observed for all three peptides, but with a significantly weaker maximum peak intensity for **StAMP-8** compared to **StAMP-9-10**. The increasing light emission was followed by a continuing decrease in RLU. Compared to the control chlorhexidine, a bacterial agent known for its membrane disruptive properties [47], **StAMP-8-10** required a longer reaction time before disrupting the membrane. Chlorhexidine acetate has a molecular weight of 625.5 g/mol, making the concentration 25 µg/mL (40 µM) most comparable with the highest tested concentration for the peptides (50 µg/mL ~ 33–39 µM). As shown in Figure 3, it took approximately 30 s before a decrease in light intensity was observed in *B. subtilis* after adding **StAMP-8-10**. **StAMP-8** affected the membrane at 25 µg/mL (Figure S1), **StAMP-9** at 12.5 µg/mL (Figure S2), and **StAMP-10** affected the membrane down to 6.3 µg/mL (Figure S3). These results strongly suggest that both chlorhexidine and **StAMP-8-10** had the bacterial membrane of *B. subtilis* as their main target, but the molecular mechanisms leading to membrane disruption might be different.

**Figure 4:**
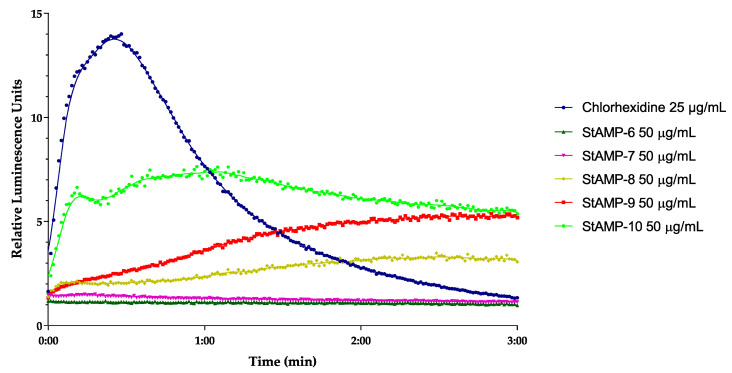
Kinetics of the antimicrobial effect on membrane integrity as measured by relative luminescence emission in *E. coli* K12 (pCSS962) in presence of D-luciferin. **StAMP-6-10** and the reference antimicrobial agent chlorhexidine was added to the bacteria. Chlorhexidine and water were used as positive and negative control. Each datapoint is the mean of three independent measurements normalized to the water control.

**StAMP-6-7** had no effect on light emission at any of the concentrations tested when looking at the membrane integrity of *E. coli* (data not shown). **StAMP-8-10** affected the bacterial membrane of *E. coli* K12 at 50 µg/mL. As shown in Figure 4, all three peptides increased the light emission, but their membrane integrity effect on the *E. coli* was less prominent than the effect on *B. subtilis* when considering the decrease in light over time. The light emission was slowly decreasing after 1 min of **StAMP-10** exposure, but when exposed to **StAMP-8** and **StAMP-9** the light emission was not decreasing within the measured time period. **StAMP-8-9** influenced the *E. coli* membrane down to 25 µg/mL (Figures S4 and S5), and **StAMP-10** at 12.5 µg/mL (Figure S6).

**Figure 5:**
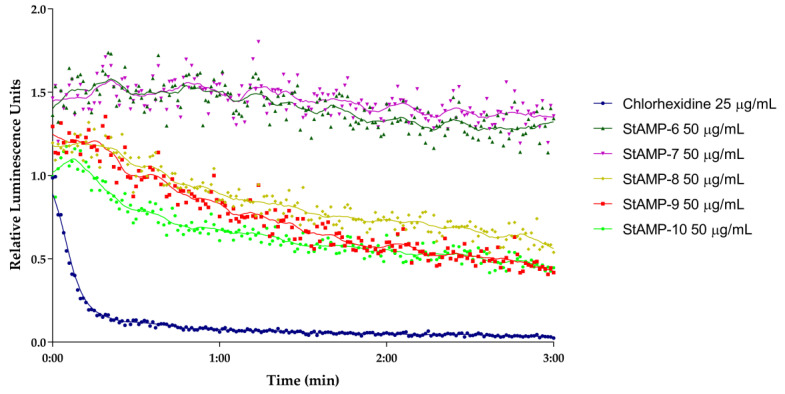
Kinetics of the antimicrobial effect on viability of *B. subtilis* 168 as measured by relative luminescence emission from the luxABCDE operon after adding **StAMP-6-10** to the bacteria. Chlorhexidine served as a positive control and water as a negative control. Each datapoint was the mean of three independent measurements normalized to the negative control.

**Figure 6:**
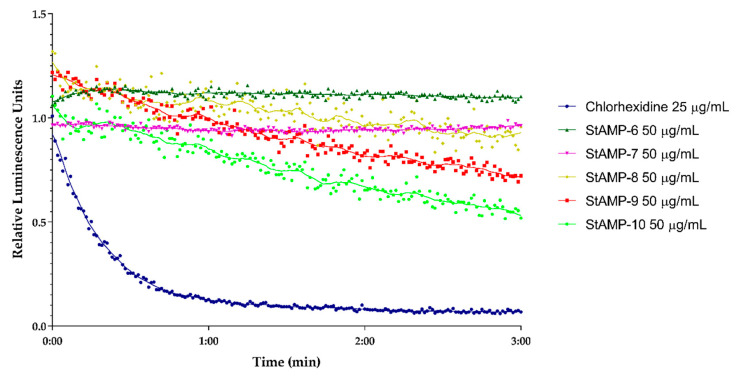
Kinetics of the antimicrobial effect on viability of *E. coli* K12 (pCGLS-11) as measured by relative luminescence emission from the *lux*CDABE operon after adding **StAMP-6-10**. Chlorhexidine served as a positive control and water as a negative control. Each datapoint was the mean of three independent measurements normalized to the negative control.

A real-time cell viability assay was used to investigate the bactericidal effect of **StAMP-6-10**. *B. subtilis* 168 contains a chromosomally integrated *lux* operon, and *E. coli* K12 a plasmid-borne *lux* operon. Both strains emit light as long as they have an active metabolism. If a compound reduces the metabolic activity of the bacteria it will reduce the viability of the cells. The results of the viability assay are shown in Figure 5 and Figure 6. These results independently confirmed the bactericidal effect observed in the membrane integrity assays. **StAMP-6-7** had a minor effect on the viability of both *B. subtilis* and *E. coli* at 50 µg/mL. The decrease in light emission caused by **StAMP-8-10** shown in both assays confirmed that these three peptides killed both strains at 50 µg/mL, but not as rapidly as the control chlorhexidine. The same activity observed for **StAMP-8-10** in the membrane integrity assay was also observed in both viability assays (Figures S7–S12). Chlorhexidine showed a dose-dependent activity in both integrity and viability assay against both bacteria tested (Figures S13–S16). Only at the lowest concentration tested (0.8 µg/mL), was there no observed membrane activity for these assays.

### 2.3. Antifungal Activity

The synthesized peptides were subjected to antifungal screening against the molds *Aureobasidium pullulans* and *Rhodotorula* sp., and the yeast *Candida albicans* (Table 3). The first lead peptide **StAMP-1** was inactive (MIC > 250 µg/mL) against all three fungal strains tested. As observed during the antibacterial screening, increased hydrophobicity of the peptides (due to Trp substitutions) generally increased the antifungal activity, with **StAMP-8** being the most potent peptide (MIC = 7.8–15.6 µg/mL) in this mutant series. In contrast to antibacterial activity, replacement of all Lys residues in **StAMP-7** with Arg, resulting in **StAMP-9,** did not improve the antifungal activity against the three fungal strains tested. Fungal membranes are more zwitterionic compared to the negatively charged bacterial membranes [48], and these differences naturally could make the membranes vulnerable to different antimicrobials.

### 2.4. Hemolytic, Cytotoxic and Anti-Inflammatory Properties

The peptides (**StAMP-1-11)** were assayed for hemolytic activity against sheep red blood cells, cytotoxic activity against the human melanoma cell line A2058 and the non-malignant human lung fibroblast cell line MRC-5, and their ability to inhibit LPS induced TNF-α production by the human acute leukemia monocytic THP-1 cell line. None of the synthesized peptides displayed any hemolytic activity (<1% hemolysis) against sheep red blood cells at concentrations up to 250 µg/mL. No cytotoxic or anti-inflammatory activities were detected for any of the peptides, even at the highest concentration tested (100 µg/mL). The results from these assays indicate that the peptides may be well tolerated in an in vivo setting. 

## 3. Materials and Methods

### 3.1. Sequence Analysis and Peptide Design

The 36 amino acids sequence of turgencin A, a Cys-rich AMP isolated from the marine ascidian *S. turgens* [24], served as a starting point for the sequence analysis. All Cys residues were replaced by Ala prior to in silico prediction of antimicrobial potential of 10-residue sequences of the linear version of turgencin A, using the online prediction tool on the Collection of Anti-Microbial Peptides (CAMP_R3_) server (http://www.camp3.bicnirrh.res.in/predict/) [25]. A sliding window strategy (using a window size of ten amino acid residues) was used to locate putative AMP stretches within the full peptide sequence. The peptide segments were analyzed by means of four prediction models: support vector machines (SVM), random forests (RF), artificial neural network (ANN), and discriminant analysis (DA). The antimicrobial potential of the peptides were also predicted using the SVM model in a database of antimicrobial peptides (ADAM) (http://bioinformatics.cs.ntou.edu.tw/ADAM/svm_tool.html) [26]. Physicochemical properties and primary sequence homology to known AMPs were investigated using the calculator, predictor, and BLAST tools of the antimicrobial peptide database (APD3) (http://aps.unmc.edu/AP/) [40]. The synthesized peptides were named with the acronym StAMP (*S. turgens* antimicrobial peptide) followed by a progressive number. The peptide sequence with the highest overall predicted AMP score from all models was the sequence GKKPGGWKAK, which became our first lead peptide **StAMP-1** and basis for further studies. As for turgencin A, the lead peptide **StAMP-1** and the following peptides synthesized were C-terminally amidated to increase the overall net-positive charge of the peptide series. In order to improve antimicrobial activity, an amino acid modification and replacement strategy was chosen for a set of derivatives. Firstly, a Trp enrichment strategy within the central core (PGGW) of the lead peptide was chosen to increase the overall hydrophobicity. Secondly, all four Lys residues were substituted by Arg, and finally a peptide was made with the Trp residues replaced by Leu. All designed StAMPs were subjected to AMP prediction to validate the in silico models used and described above (Table S1 in the SI).

### 3.2. Peptide Synthesis

The peptides were synthesized by microwave assisted fluorenylmethoxycarbonyl solid-phase peptide synthesis (Fmoc-SPSS), using a Biotage^®^ Initiator+Alstra™ (Uppsala, Sweden) fully automated peptide synthesizer. Fmoc-amino acids and solvents were purchased from Sigma-Aldrich (St. Louis, MO, USA) whereas Rink amide ChemMatrix resin was obtained from Biotage.

Rink Amide ChemMatrix resin (loading 0.44–0.48 mmol/g) was used to obtain peptides with an amidated C-terminus and each synthesis was scaled to 0.165 mmol. The resin was initially swelled at 70 °C for 20 min. Peptide synthesis was performed by coupling the Fmoc-amino acids (0.5 M in DMF, 4 equiv.) using the coupling reagent O-(6-chlorobenzotriazol-1-yl)-*N,N,N′,N′*-tetramethyluronium hexafluorophosphate (HCTU, 0.6 M in *N,N*-dimethylformamide (DMF), 3.92 equiv.), and the base *N,N*-diisopropylethylamine (DIEA, 2.0 M in *N*-methylpyrrolidone (NMP), 8 equiv.). The coupling reactions were performed with microwave heating (75 °C) and coupling times of 5 min. Coupling of Fmoc-Arg(Pbf)-OH (0.5 M in DMF, 4 equiv.) was done at room temperature for 60 min for peptides **StAMP-9**, **-10** and **-11** and the coupling time of the remaining Fmoc-amino acids was increased from 5 to 10 min (microwave heating, 75 °C) due to the high lipophilicity of the fully side-chain protected peptides. After each coupling step, the temporary Fmoc-protecting group was cleaved using a solution of 20% piperidine in DMF (4.5 mL) at room temperature for 3 min and then repeated for 10 min. When the synthesis was completed the resin with peptide attached was washed with dichloromethane (DCM, 4.5 mL, 6 times, 45 s), followed by washing the resin with diethyl ether (4.5 mL, 3–4 times) using a vacuum manifold, and then dried in a vacuum desiccator overnight. The removal of protecting groups and cleaving the peptide from the resin was performed using a cleavage cocktail consisting of 95% trifluoroacetic acid (TFA, Sigma-Aldrich), 2.5% triisopropyl silane (TIS, Sigma-Aldrich), and 2.5% H_2_O (total volume 10 mL) for 1 h and then repeated for 3 h. A vacuum manifold was used to isolate the cleaved peptide solution by filtration. The peptide filtrates were pooled, and the volume reduced in vacuo before the crude peptide was precipitated by the addition of ice-cold diethyl ether. The ether solution was decanted, and the procedure repeated twice by washing with ice cold diethyl ether. After the final decantation, the precipitated crude peptide was dried in vacuo prior to purification.

### 3.3. Peptide Purification and Verification

The synthesized peptides were purified by reversed-phase high-performance liquid chromatography (RP-HPLC) using a Waters preparative HPLC system equipped with a photodiode array (PDA) detector and an XBridge C_18_, 5 µm, 10 × 250 mm column (Waters Associates, Milford, MA, USA). The separation was performed using linear gradients of acetonitrile (95% in water) and water, and both eluents containing 0.1% TFA (Sigma-Aldrich) with a flow rate of 10 mL/min. The purity of the peptides (>95%) was determined by an analytical UPLC-PDA system using an Acquity C_18_, 1.7 µm, 2.1 × 50 mm column (Waters) with the same conditions as described above, but with a flow rate of 0.6 mL/min. Molecular weight and purity of the peptides (Table S2) were confirmed using a high-resolution 6540B quadrupole time-of-flight (Q-ToF) mass spectrometer with a dual electrospray ionization (ESI) source, coupled to a 1290 Infinity UHPLC system, controlled by MassHunter software (Agilent, Santa Clara, CA, USA). The peptides were separated using a Zorbax Eclipse Plus C_18_, 1.8 µm, 2.1 × 50 mm column (Agilent). A gradient running from 3–20% acetonitrile containing 0.1% formic acid over 15 min with a flow rate of 0.4 mL/min was applied for the determination of the hydrophobicity (retention times) of the peptides.

### 3.4. Antibacterial Assay (Growth Inhibition)

The synthesized peptides were screened for antibacterial activity against five strains of G+ bacteria: *B. subtilis* (Bs, ATCC 23857), *C. glutamicum* (Cg, ATCC 13032), *S. aureus* (Sa, ATCC 9144), *Micrococcus luteus* (Ml), *B. megaterium* (Bm) (the last two were obtained from professor Olaf B. Styrvold, UiT The Arctic University of Norway). 

Two strains of G- bacteria, *P. aeruginosa* (Pa, ATCC 27853) and *E. coli* (Ec, ATCC 25922), were also used. Cultures stored at -80 °C in glycerol were transferred to Müller–Hinton plates (MH, Difco Laboratories, Detroit, MI, USA) and incubated for 24 h at 35 °C. Colonies of each strain were transferred to 5 mL liquid MH medium and left shaking (600 rpm) at room temperature overnight. An aliquot of actively growing bacteria (20 µL) was inoculated in 5 mL MH medium and left shaking for 2 h at room temperature. In order to have a sensitive bioassay, the bacterial cultures were diluted with medium to only 2.5–3 × 10^4^ bacteria/mL and an aliquot of 50 µL was added to each well in 96 microwell plates (Thermo Fisher Scientific, Roskilde, Denmark) preloaded with peptide solution (50 µL). The antibacterial assays were performed as previously described [49]. The microtiter plates were incubated for 24 h at 35 °C with optical density (595 nm) recorded every hour using an Envision 2103 multilabel reader, controlled by a Wallac Envision manager (PerkinElmer, Waltham, MA, USA). The minimum inhibitory concentration (MIC) was defined as the sample concentration showing an optical density less than 10% of the negative (growth) control, consisting of bacteria and MQ-H_2_O. Oxytetracycline (concentrations ranging from 20–0.02 µg/mL, Sigma-Aldrich) and indolicidin (concentration ranging from 200–0.2 µg/mL, Sigma-Aldrich) served as a positive (inhibition) control. The synthetic peptides were tested for antibacterial activity in concentrations ranging from 250 to 0.5 µg/mL in two-fold dilutions. All tests were performed in triplicates.

A killing experiment was performed on **StAMP-9** by using actively growing cultures of *B. subtilis* (ATCC 23857) and *E. coli*. (ATCC 25922). The procedure was performed as previously described [50]. Both tests were performed in triplicates.

### 3.5. Real-Time Assay Measuring Immediate Bacteria Membrane Disruption

The real-time bacterial membrane integrity assay was performed using *B. subtilis* 168 (ATCC 23857) and *E. coli* K12 (ATCC MC1061), both carrying the plasmid pCSS962 with the eukaryotic luciferase gene *lucGR*. Luciferase is dependent on D-luciferin as substrate to emit light, a substrate that does not penetrate intact cell membranes. The assay is a modification of a previously described protocol [44] and was conducted as previously described [24]. The bacteria (~5 × 10^7^ bacteria/mL) were subjected to ranging concentrations of **StAMP-6-10** (50–3.1 µg/mL) and the positive control to chlorhexidine acetate (assay concentrations 100–1.6 µg/mL, Fresenius Kabi, Halden, Norway). Three independent measurements were conducted, and measurements were normalized to the untreated water controls.

### 3.6. Real-Time Assay Measuring Immediate Bacterial Cell Viability

A real-time cell viability assay (modified from [51]) was performed using *B. subtilis* 168 carrying an optimized *lux*ABCDE operon controlled by the promoter P*veg* [52], and *E. coli* K12 carrying the plasmid pCGLS-11 [53] with the *lux*CDABE operon from *Xenorhabdus luminescence*. The procedure was conducted as previously described [24]. The bacteria (~5 × 10^7^ bacteria/mL) were subjected to ranging concentrations of **StAMP-6-10** (50–3.1 µg/mL) and the positive control to chlorhexidine acetate (assay concentrations 100–1.6 µg/mL). MQ-H_2_O was used as a negative control. Three independent measurements were conducted, and measurements were normalized to the untreated water controls.

### 3.7. Antifungal Assay

**StAMP-1–11** were screened for antifungal activity against *C. albicans* (Ca, ATCC 10231), *A. pullulans* (Ap) and *Rhodotorula* sp. (Rh) (the last two were obtained from professor Arne Tronsmo, The Norwegian University of Life Sciences, Ås, Norway). The antifungal assay was performed as previously described [54] with a few modifications. Briefly, fungal spores were dissolved in potato dextrose broth (Difco Laboratories) with 2% D(+)-glucose (Merck, Darmstadt, Germany) to a concentration of 4 × 10^5^ spores/mL. The spores (50 µL) were inoculated on 96 microwell plates (Thermo Fisher Scientific) containing the synthetic peptides (50 µL) and controls (water or antibiotic). The peptides were diluted in MQ-H_2_O at final concentrations ranging from 250–3.9 µg/mL in two-fold serial dilutions. Triclosan (Sigma-Aldrich, Steinheim, Germany) was used as a positive (antifungal) control (32–0.25 µg/mL), and MQ-H_2_O as a negative (growth) control. Cultures were grown in room temperature for 24 h (Ca) and 48 h (Ap and Rh). Growth inhibition was determined by measuring OD values at 600 nm by a microplate reader (Synergy H1 Hybrid Multi-Mode Reader, BioTek, Winooski, VT, USA). The MIC values were defined as the lowest concentration of the peptides that showed >90% inhibition compared to the negative growth control (as measured by OD). All experiments were done in triplicate.

### 3.8. Hemolytic Activity Assay

The hemolytic activity assays were performed as previously described [23], but instead of using human red blood cells, defibrinated sheep blood (Thermo Scientific, No. R54016) was used. Briefly, the blood was centrifuged (450× *g*) for 10 min, the supernatant removed, and the pellet dissolved in phosphate-buffered saline (PBS; 320 mOSM, pH 7.4). This was done three times before the pellet was adjusted in PBS to a suspension containing 10% red blood cells (RBC). An aliquot of 10 µL of the RBC suspension was added to each well in 96 microwell plates with round bottom (Nunc, Thermo Fisher Scientific), preloaded with 90 µL of the synthetic peptides and controls (PBS or Triton). The peptides were diluted in PBS at final concentrations ranging from 250–2 µg/mL in twofold dilutions. As a positive (hemolysis) control, Triton X-100 (Sigma-Aldrich, Saint Louis, MO, USA) was used at a final concentration of 0.05%, and PBS was used as a negative control. The plates were incubated at 37 °C for 1 h on a shaker, and afterwards centrifuged at 450× *g* for 10 min. The supernatants were transferred to 96 microwell plates with flat bottom (Thermo Fisher Scientific), and the absorbance was measured at 550 nm. The percent hemolysis was calculated using the formula [(A_sample_ − A_baseline_)/(A_triton_ − A_baseline_)] × 100, where the PBS was used as baseline and Triton X-100 (Sigma-Aldrich) as 100% hemolysis. The experiment was performed in triplicate.

### 3.9. Human Cell Viability Assay

The human melanoma cell line A2058 (ATTC CRL-11144) and the non-malignant human lung fibroblast cell line MRC-5 (ATTC CCL-171) were assayed for sensitivity against **StAMP-1-11** at ranging concentrations between 100 and 5 µg/mL in a two-fold dilution series. The assays were performed as previously described [55]. Both assays were performed in triplicate in two independent experiments.

### 3.10. Anti-Inflammatory Activity Assay 

The ability of **StAMP-1-11** to inhibit LPS induced TNF-α production by the human acute leukemia monocytic THP-1 cell line (ATCC TIB-202) was assayed as previously described [56]. Cells were added with 100 µg/mL of **StAMP-1-11**. The experiment was conducted in triplicate in two independent experiments.

## 4. Conclusions

The overall most potent antimicrobial peptide **StAMP-9** has several advantages, including potent antimicrobial activity, immediate effect on bacterial membranes, and high selectivity (non-hemolytic, non-cytotoxic), and it has a short sequence consisting of 10 natural amino acids. Although **StAMP-9** might be prone to proteolytic digestion, its simple sequence should facilitate rapid production, at low cost, and accelerate further studies and development into a clinical drug candidate. Proteolytic resistance might be improved by the insertion of D-amino acids or other chemically modified amino acids, or by cyclization [57]. This study also illustrates the potential for combining web-based and computational resources with a rational design of short antimicrobial peptides derived from larger peptides or proteins of natural origin. Future studies should be aimed at checking and/or improving the proteolytic stability of StAMP-9 as well as studying its efficacy in vivo, for instance, in a mouse infection model.

## Figures and Tables

**Table 1:** Characteristics and in silico antimicrobial activity prediction of 10-mer peptide sequences modelled from Turgencin A_lin_; a linear version of turgencin A where the Cys residues were replaced by Ala. The highlighted sequence 18–27 (GKKPGGWKAK) in bold was the only sequence that was predicted to be antimicrobial by all four in silico models.

Peptide Region	Sequence	Net Charge	Hydro-Phobic Ratio (%)	Boman Index (kcal/mol)	CAMP_R3_^1^				ADAM ^2^
					SVM	RF	ANN	DA	SVM
1–10	GPKTKAAAKM	+3	40	1.04	1.000	0.479	AMP	0.681	1.49
2–11	PKTKAAAKMA	+3	50	0.96	0.548	0.439	NAMP	0.343	1.95
3–12	KTKAAAKMAA	+3	60	0.78	0.131	0.443	AMP	0.325	2.53
4–13	TKAAAKMAAK	+3	60	0.78	0.972	0.439	AMP	0.170	2.53
5–14	KAAAKMAAKL	+3	70	0.03	0.478	0.428	AMP	0.797	2.59
6–15	AAAKMAAKLA	+2	80	−0.70	0.980	0.363	AMP	0.785	2.64
7–16	AAKMAAKLAT	+2	70	−0.26	0.989	0.358	AMP	0.483	2.41
8–17	AKMAAKLATA	+2	70	−0.26	0.947	0.325	AMP	0.312	2.41
9–18	KMAAKLATAG	+2	60	−0.17	0.330	0.281	AMP	0.254	2.13
10–19	MAAKLATAGK	+2	60	−0.17	0.651	0.270	AMP	0.135	2.13
11–20	AAKLATAGKK	+3	50	0.61	0.615	0.425	AMP	0.786	2.07
12–21	AKLATAGKKP	+3	40	0.79	0.751	0.376	AMP	0.535	1.29
13–22	KLATAGKKPG	+3	30	0.87	0.244	0.377	AMP	0.647	1.58
14–23	LATAGKKPGG	+2	30	0.23	0.736	0.379	AMP	0.649	2.16
15–24	ATAGKKPGGW	+2	30	0.49	0.075	0.282	AMP	0.781	2.52
16–25	TAGKKPGGWK	+3	20	1.22	0.880	0.398	AMP	0.591	2.53
17–26	AGKKPGGWKA	+3	30	0.78	0.490	0.427	AMP	0.930	2.85
18–27	**GKKPGGWKAK**	+4	20	1.52	0.968	0.559	AMP	0.884	2.85
19–28	KKPGGWKAKL	+4	30	1.12	0.165	0.566	AMP	0.815	2.61
20–29	KPGGWKAKLA	+3	40	0.38	0.027	0.448	AMP	0.689	2.44
21–30	PGGWKAKLAE	+1	40	0.51	0.017	0.190	AMP	0.018	2.00
22–31	GGWKAKLAEL	+1	50	0.02	0.325	0.238	AMP	0.041	2.37
23–32	GWKAKLAELG	+1	50	0.02	0.444	0.241	AMP	0.041	2.37
24–33	WKAKLAELGA	+1	60	0.21	0.205	0.252	NAMP	0.024	2.04
25–34	KAKLAELGAD	0	50	0.00	0.004	0.293	NAMP	0.002	1.47
26–35	AKLAELGADA	−1	60	0.28	0.281	0.329	NAMP	0.003	1.44
27–36	KLAELGADAV	−1	60	0.80	0.799	0.373	NAMP	0.007	0.56

^1^ CAMP_R3_: collection of anti-microbial peptides; SVM: support vector machines; RF: random forests; ANN: artificial neural networks; and DA: discriminant analysis. ^2^ ADAM: a database of AMPs.

**Table 2:** Sequences and physicochemical properties of the synthesized **StAMP-1-11** peptides. **StAMP-1** corresponds to the C-terminal amidated first lead peptide sequence 18–27.

Peptide	Sequence ^1^	Monoisotopic Mass (Da)		Net Charge	Boman Index (kcal/mol)	Hydro-Phobic Ratio (%)	Rt ^3^
		Theoretical	Measured ^2^				
StAMP-1	GKKPGGWKAK-NH2	1054.64	1054.64	+5	1.52	20	0.40
StAMP-2	GKK**W**GGWKAK-NH2	1143.67	1143.67	+5	1.29	30	1.75
StAMP-3	GKKP**W**GWKAK-NH2	1183.70	1183.70	+5	1.38	30	2.17
StAMP-4	GKKPG**W**WKAK-NH2	1183.70	1183.70	+5	1.38	30	2.05
StAMP-5	GKK**WW**GWKAK-NH2	1272.72	1272.72	+5	1.15	40	5.21
StAMP-6	GKK**W**G**W**WKAK-NH2	1272.72	1272.72	+5	1.15	40	5.39
StAMP-7	GKKP**WW**WKAK-NH2	1312.76	1312.76	+5	1.24	40	5.70
StAMP-8	GKK**WWW**WKAK-NH2	1401.78	1401.78	+5	1.01	50	8.77
StAMP-9	G**RR**P**WW**W**R**A**R**-NH2	1424.78	1424.78	+5	4.99	40	6.65
StAMP-10	G**RRWWW**W**R**A**R**-NH2	1513.81	1513.81	+5	4.76	50	9.20
StAMP-11	G**RR**P**LLLR**A**R**-NH2	1205.79	1205.79	+5	4.21	40	2.54

^1^ Amino acid substitutions are shown in bold, ^2^ Measured by high-resolution mass spectrometry, ^3^ Retention time (min) on an analytical RP-HPLC C_18_-column using a fixed mobile phase gradient.

**Table 3:** Antimicrobial activities of turgencin A and the synthesized **StAMP-1–11** peptides.

Antimicrobial Activity (MIC; µg/mL) ^1^										
	Gram-Pos					Gram-Neg		Fungi		
Peptide	Bm	Bs	Cg	Ml	Sa	Ec	Pa	Ap	Ca	Rh
Turgencin A ^2^	0.5	1.5	1.5	8.0	23.3	3.0	5.9	92.6	46.3	23.2
StAMP-1	250	>250	250	>250	>250	>250	>250	>250	>250	>250
StAMP-2	3.9	125	31.3	250	>250	>250	>250	62.5	125	62.5
StAMP-3	3.9	>250	15.6	250	>250	>250	>250	62.5	125	62.5
StAMP-4	3.9	125	3.9	125	>250	>250	>250	62.5	62.5	31.3
StAMP-5	1.0	15.6	2.0	15.6	>250	31.3	250	31.3	31.3	15.6
StAMP-6	1.0	3.9	3.9	62.5	250	62.5	>250	62.5	62.5	31.3
StAMP-7	1.0	3.9	2.0	31.3	125	31.3	250	15.6	31.3	15.6
StAMP-8	3.9	7.8	7.8	15.6	125	62.5	125	7.8	15.6	15.6
StAMP-9	1.0	3.9	2.0	3.9	7.8	7.8	31.3	31.3	31.3	15.6
StAMP-10	3.9	7.8	7.8	15.6	62.5	15.6	31.3	62.5	62.5	15.6
StAMP-11	7.8	>250	31.3	62.5	>250	>250	>250	250	125	31.3
Indolicidin	3.1	6.3	1.6	12.5	12.5	25.0	>250	25.0	100	25.0
Oxytetracycline	0.6	10.0	0.2	1.3	0.04	1.3	2.5	n.t ^3^	n.t	n.t.
Triclosan	n.t	n.t	n.t	n.t	n.t	n.t	n.t	3.1	3.1	1.6

^1^ Microbial strains: Bm—*Bacillus megaterium*, Bs—*Bacillus subtilis*, Cg—*Corynebacterium glutamicum*, Ml—*Micrococcus luteus*, Sa—*Staphylococcus aureus*, Ec—*Escherichia coli*, Pa—*Pseudomonas aeruginosa*, Ap—*Aurobasidium pollulans*, Ca—*Candida albicans*, Rh—*Rhodotorula* sp. ^2^ Antibacterial data for turgencin A against Bs, Cg, Sa, Ec and Pa are derived from Hansen et al. [24]. ^3^ nt: Not tested.

## Supplementary Materials

The following are available online at https://www.mdpi.com/1422-0067/21/15/5460/s1, Figure S1. Antimicrobial effect on membrane integrity as measured by RLU in *B. subtilis* (pCSS962) treated with chlorhexidine and different concentration of **StAMP-8**, Figure S2. Antimicrobial effect on membrane integrity as measured by RLU in *B. subtilis* (pCSS962) treated with chlorhexidine and different concentration of **StAMP-9**, Figure S3. Antimicrobial effect on membrane integrity as measured by RLU in *B. subtilis* (pCSS962) treated with chlorhexidine and different concentration of **StAMP-10**, Figure S4. Antimicrobial effect on membrane integrity as measured by RLU in *E. coli* (pCSS962) treated with chlorhexidine and different concentration of **StAMP-8**, Figure S5. Antimicrobial effect on membrane integrity as measured by RLU in *E. coli* (pCSS962) treated with chlorhexidine and different concentration of **StAMP-9**, Figure S6. Antimicrobial effect on membrane integrity as measured by RLU in *E. coli* (pCSS962) treated with chlorhexidine and different concentration of **StAMP-10**, Figure S7. Antimicrobial effect on viability as measured by RLU in *B. subtilis* (pCGLS-11) treated with chlorhexidine and different concentration of **StAMP-8**, Figure S8. Antimicrobial effect on viability as measured by RLU in *B. subtilis* (pCGLS-11) treated with chlorhexidine and different concentration of **StAMP-9**, Figure S9. Antimicrobial effect on viability as measured by RLU in *B. subtilis* (pCGLS-11) treated with chlorhexidine and different concentration of **StAMP-10**, Figure S10. Antimicrobial effect on viability as measured by RLU in *E. coli* (pCGLS-11) treated with chlorhexidine and different concentration of **StAMP-8**, Figure S11. Antimicrobial effect on viability as measured by RLU in *E. coli* (pCGLS-11) treated with chlorhexidine and different concentration of **StAMP-9**, Figure S12. Antimicrobial effect on viability as measured by RLU in *E. coli* (pCGLS-11) treated with chlorhexidine and different concentration of **StAMP-10**, Figure S13. Antimicrobial effect on membrane integrity as measured by RLU in *B. subtilis* (pCSS962) treated with different concentrations of chlorhexidine, Figure S14. Antimicrobial effect on membrane integrity as measured by RLU in *E. coli* (pCSS962) treated with different concentrations of chlorhexidine, Figure S15. Antimicrobial effect on viability as measured by RLU in *B. subtilis* (pCGLS-11) treated with different concentrations of chlorhexidine, Figure S16. Antimicrobial effect on viability as measured by RLU in *E. coli* (pCGLS-11) treated with different concentrations of chlorhexidine, Table S1. Antimicrobial activity prediction of the designed StAMPs.
