# Supplementary material for: Antimicrobial Activity of Small Synthetic Peptides Based on the Marine Peptide Turgencin A: Prediction of Antimicrobial Peptide Sequences in a Natural Peptide and Strategy for Optimization of Potency
Source: Int J Mol Sci. 2020 Jul 30;21(15):5460. doi: 10.3390/ijms21155460 (PMC7432809; doi:10.3390/ijms21155460)
Supplement: Supplementary file 1 [file ijms-21-05460-s001.pdf]

Supporting information

Antimicrobial activity of small synthesized synthetic peptides based on the marine peptide turgencin A: prediction of antimicrobial peptide sequences in a natural peptide and strategy for optimization of potency

Ida K. Ø. Hansen [1,*], Tomas Lövdahl [2], Danijela Simonovic [2], Kine Ø. Hansen [3], Aron J. C. Andersen [1], Hege Devold [1], Céline S. M. Richard [1], Jeanette H. Andersen [3], Morten B. Strøm [2] and Tor Haug [1,*]

[1] Norwegian College of Fishery Science, Faculty of Biosciences, Fisheries and Economics, UiT The Arctic University of Norway, 9037 Tromsø, Norway; ajca@dtu.dk (A.J.C.A.); hege.devold@uit.no (H.D.); celine.s.richard@uit.no (C.S.M.R.)

[2] Department of Pharmacy, Faculty of Health Sciences, UiT The Arctic University of Norway, 9037 Tromsø, Norway; tlovdahl88@hotmail.com (T.L.); danijela.simonovic@uit.no (D.S.); morten.strom@uit.no (M.B.S.)

[3] Marbio, Faculty of Biosciences, Fisheries and Economics, UiT The Arctic University of Norway, Breivika, N-9037, Tromsø, Norway; kine.o.hanssen@uit.no (K.Ø.H.); jeanette.h.andersen@uit.no (J.H.A.)

Table of contents

Figure S1. Antimicrobial effect on membrane integrity as measured by RLU in *B. subtilis* (pCSS962) treated with chlorhexidine and different concentration of **StAMP-8**

Figure S2. Antimicrobial effect on membrane integrity as measured by RLU in *B. subtilis* (pCSS962) treated with chlorhexidine and different concentration of **StAMP-9**

Figure S3. Antimicrobial effect on membrane integrity as measured by RLU in *B. subtilis* (pCSS962) treated with chlorhexidine and different concentration of **StAMP-10**

Figure S4. Antimicrobial effect on membrane integrity as measured by RLU in *E. coli* (pCSS962) treated with chlorhexidine and different concentration of **StAMP-8**

Figure S5. Antimicrobial effect on membrane integrity as measured by RLU in *E. coli* (pCSS962) treated with chlorhexidine and different concentration of **StAMP-9**

Figure S6. Antimicrobial effect on membrane integrity as measured by RLU in *E. coli* (pCSS962) treated with chlorhexidine and different concentration of **StAMP-10**

Figure S7. Antimicrobial effect on viability as measured by RLU in *B. subtilis* (pCGLS-11) treated with chlorhexidine and different concentration of **StAMP-8**

Figure S8. Antimicrobial effect on viability as measured by RLU in *B. subtilis* (pCGLS-11) treated with chlorhexidine and different concentration of **StAMP-9**

Figure S9. Antimicrobial effect on viability as measured by RLU in *B. subtilis* (pCGLS-11) treated with chlorhexidine and different concentration of **StAMP-10**

Figure S10. Antimicrobial effect on viability as measured by RLU in *E. coli* (pCGLS-11) treated with chlorhexidine and different concentration of **StAMP-8**

Figure S11. Antimicrobial effect on viability as measured by RLU in *E. coli* (pCGLS-11) treated with chlorhexidine and different concentration of **StAMP-9**

Figure S12. Antimicrobial effect on viability as measured by RLU in *E. coli* (pCGLS-11) treated with chlorhexidine and different concentration of **StAMP-10**
Figure S13. Antimicrobial effect on membrane integrity as measured by RLU in *B. subtilis* (pCSS962) treated with different concentrations of chlorhexidine
Figure S14. Antimicrobial effect on membrane integrity as measured by RLU in *E. coli* (pCSS962) treated with different concentrations of chlorhexidine
Figure S15. Antimicrobial effect on viability as measured by RLU in *B. subtilis* (pCGLS-11) treated with different concentrations of chlorhexidine
Figure S16. Antimicrobial effect on viability as measured by RLU in *E. coli* (pCGLS-11) treated with different concentrations of chlorhexidine
Table S1. Antimicrobial activity prediction of the designed StAMPs
Table S2. Molecular weight and purity of the StAMPs

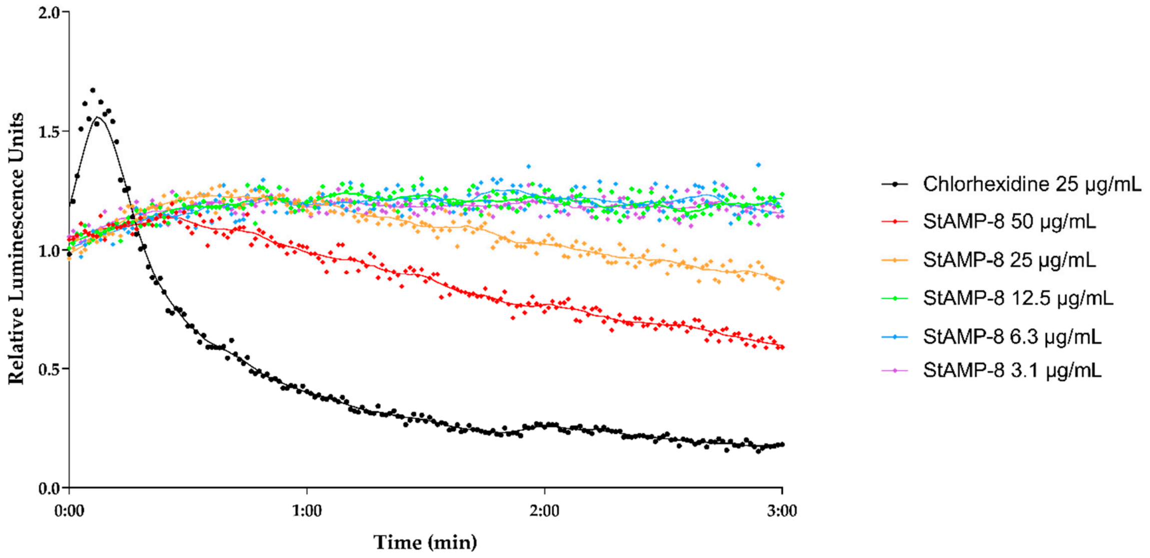

Figure S1. Kinetic of the antimicrobial effect on membrane integrity as measured by relative luminescence in *B. subtilis* (pCSS962) treated with chlorhexidine and different concentration of **StAMP-8**.

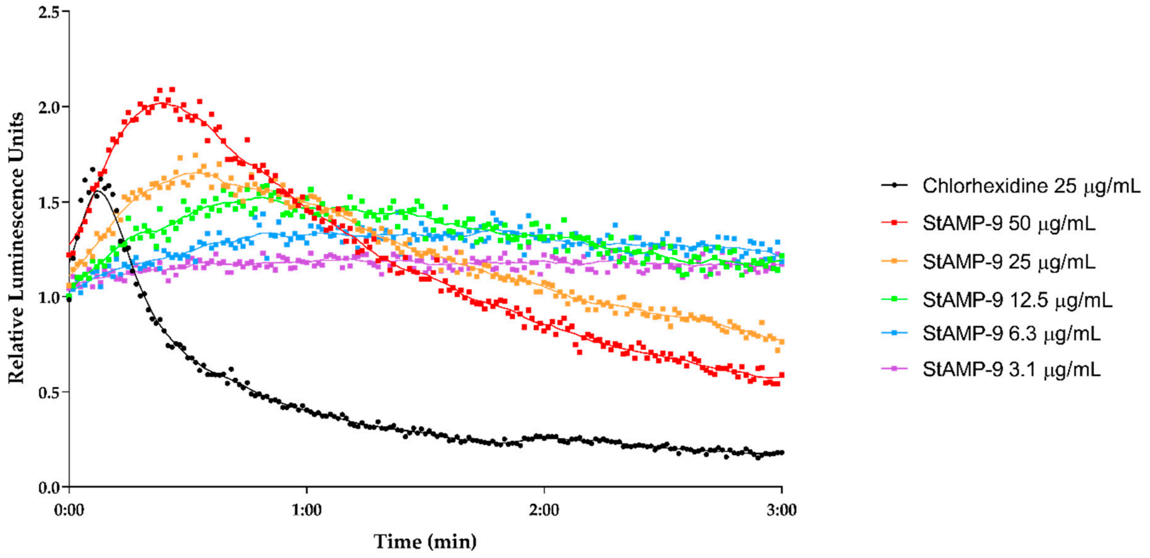

Figure S2. Kinetic of the antimicrobial effect on membrane integrity as measured by relative luminescence in *B. subtilis* (pCSS962) treated with chlorhexidine and different concentration of **StAMP-9**.

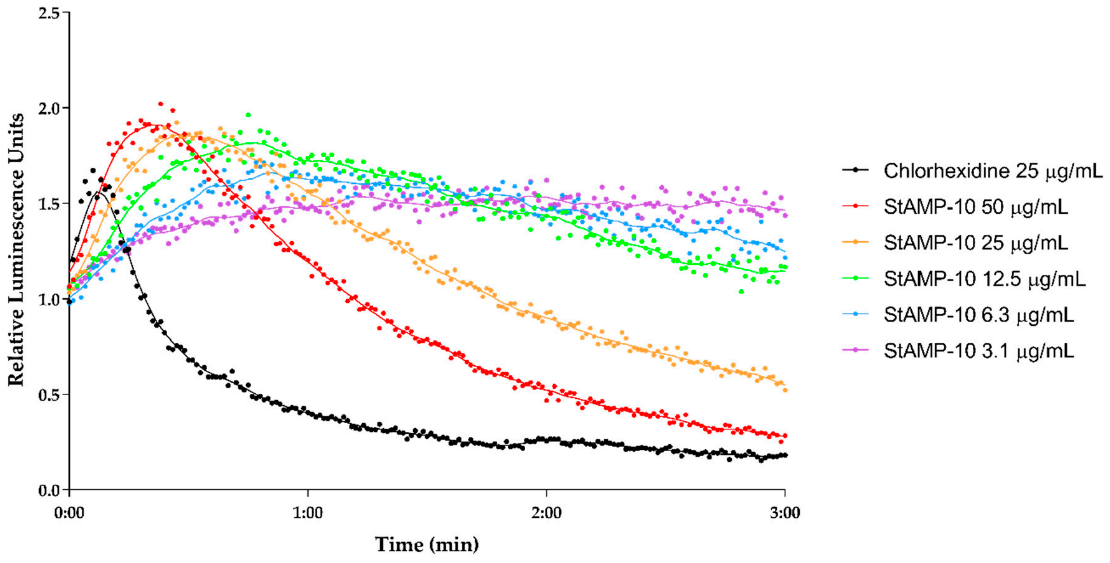

Figure S3. Kinetic of the antimicrobial effect on membrane integrity as measured by relative luminescence in *B. subtilis* (pCSS962) treated with chlorhexidine and different concentration of **StAMP-10**.

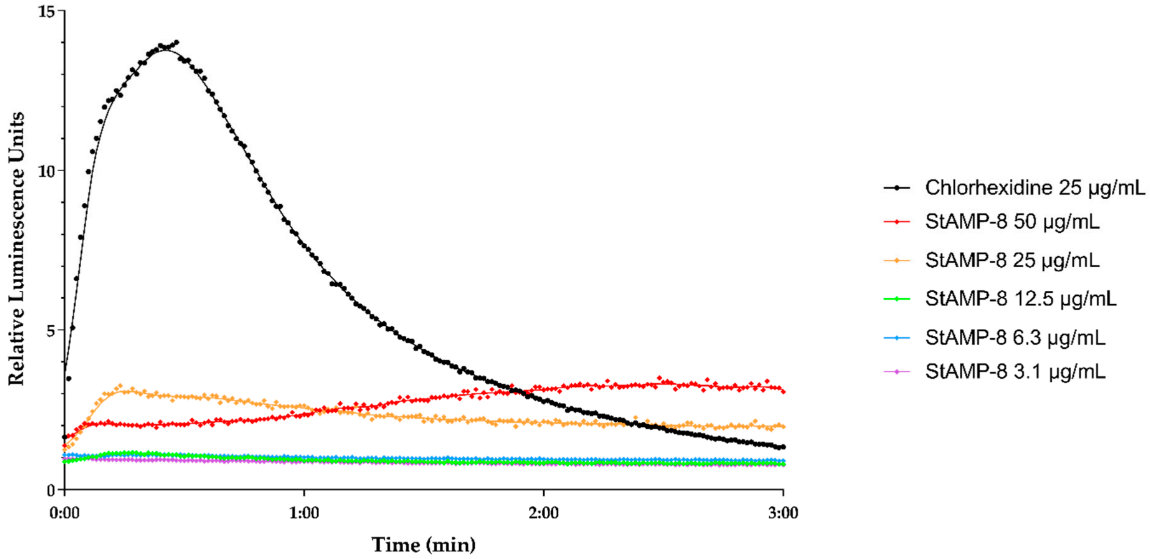

Figure S4. Kinetic of the antimicrobial effect on membrane integrity as measured by relative luminescence in *E. coli* (pCSS962) treated with chlorhexidine and different concentration of **StAMP-8**.

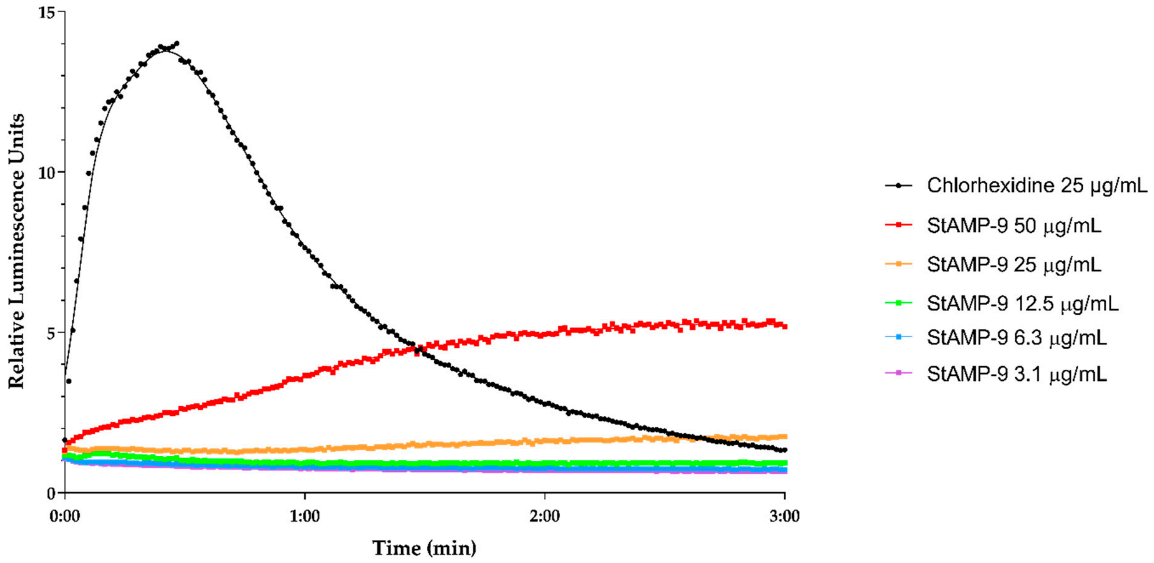

Figure S5. Kinetic of the antimicrobial effect on membrane integrity as measured by relative luminescence in *E. coli* (pCSS962) treated with chlorhexidine and different concentration of **StAMP-9**.

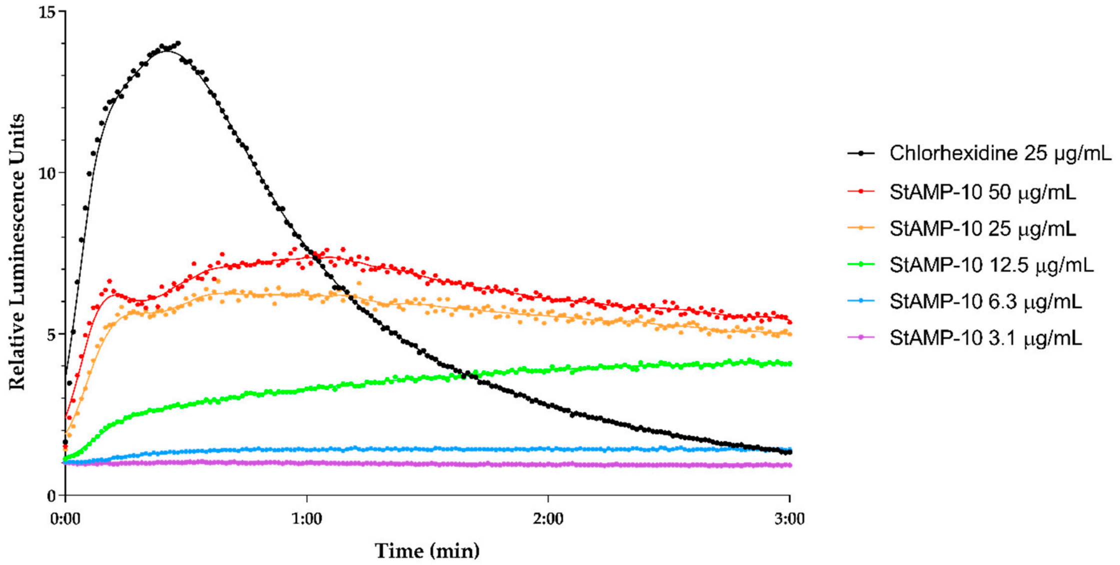

Figure S6. Kinetic of the antimicrobial effect on membrane integrity as measured by relative luminescence in *E. coli* (pCSS962) treated with chlorhexidine and different concentration of **StAMP-10**.

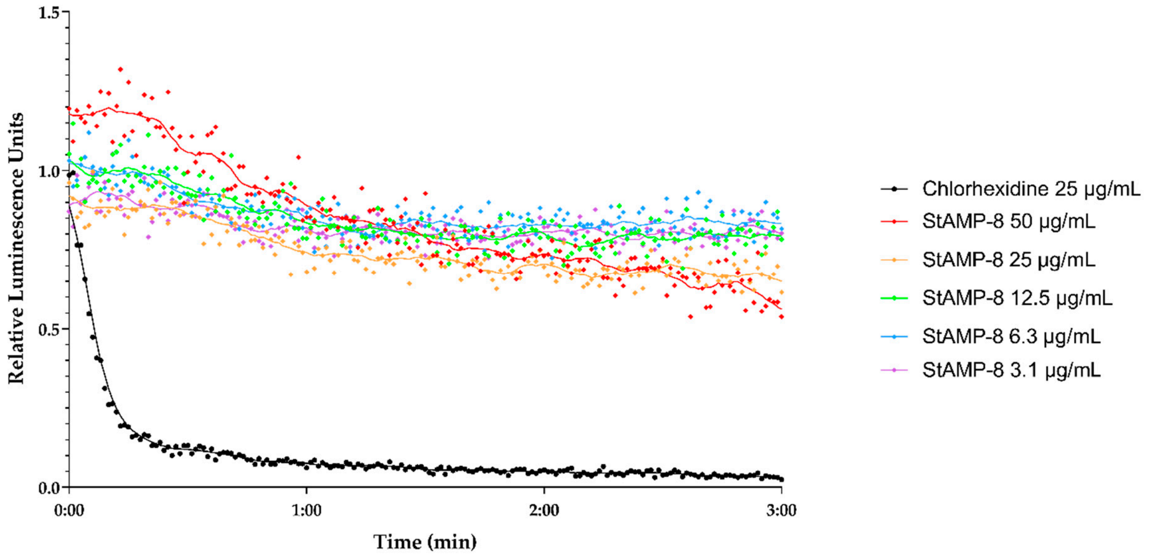

Figure S7. Kinetic of the antimicrobial effect on viability as measured by relative luminescence in *B. subtilis* (pCGLS-11) treated with chlorhexidine and different concentration of **StAMP-8**.

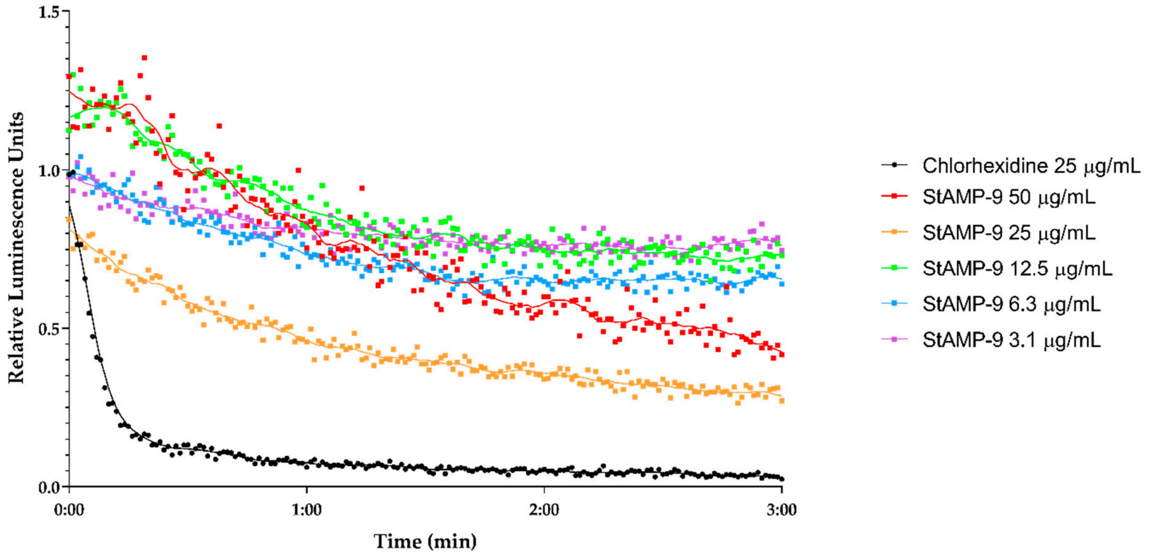

Figure S8. Kinetic of the antimicrobial effect on viability as measured by relative luminescence in *B. subtilis* (pCGLS-11) treated with chlorhexidine and different concentration of **StAMP-9**.

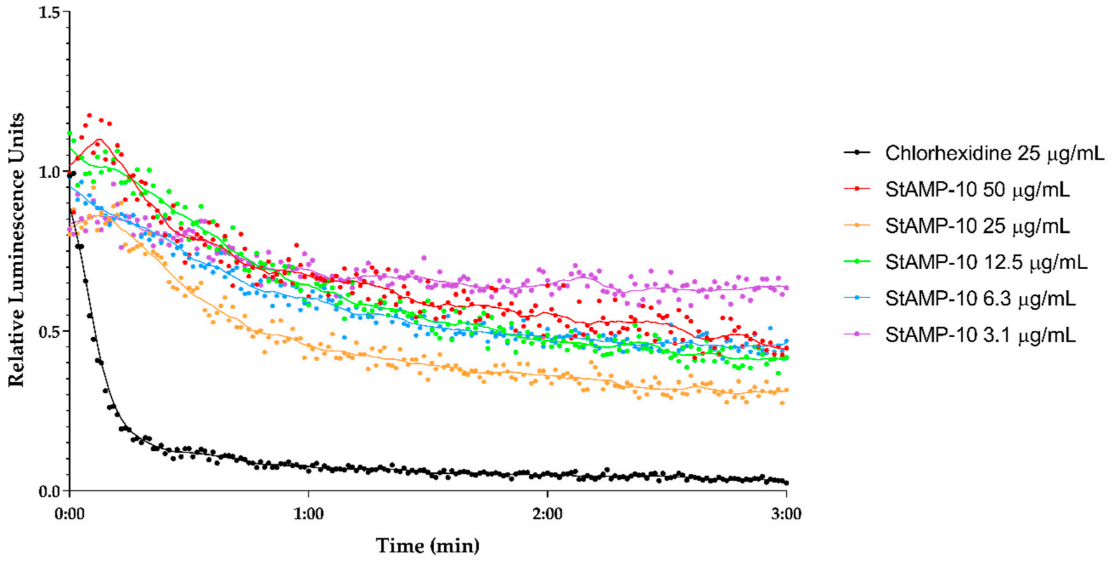

Figure S9. Kinetic of the antimicrobial effect on viability as measured by relative luminescence in *B. subtilis* (pCGLS-11) treated with chlorhexidine and different concentration of **StAMP-10**.

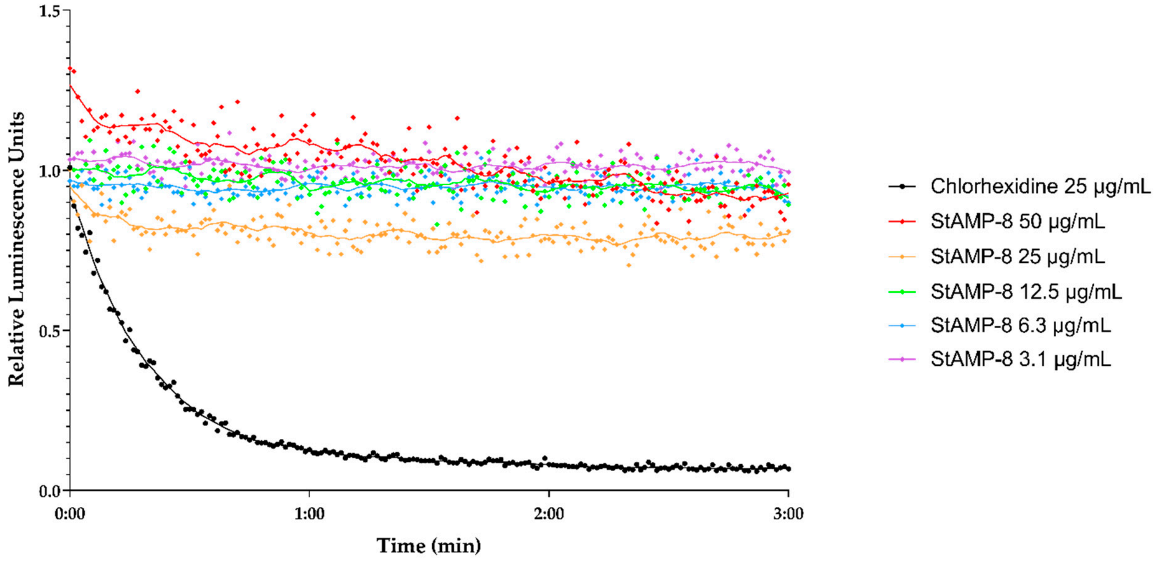

Figure S10. Kinetic of the antimicrobial effect on viability as measured by relative luminescence in *E. coli* (pCGLS-11) treated with chlorhexidine and different concentration of **StAMP-8**.

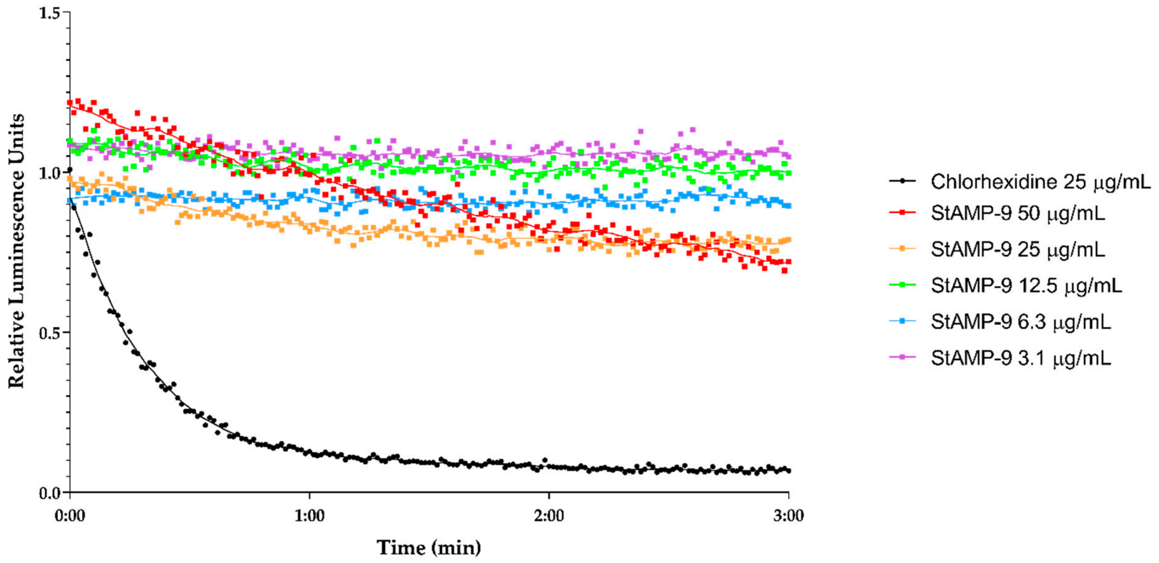

Figure S11. Kinetic of the antimicrobial effect on viability as measured by relative luminescence in *E. coli* (pCGLS-11) treated with chlorhexidine and different concentration of **StAMP-9**.

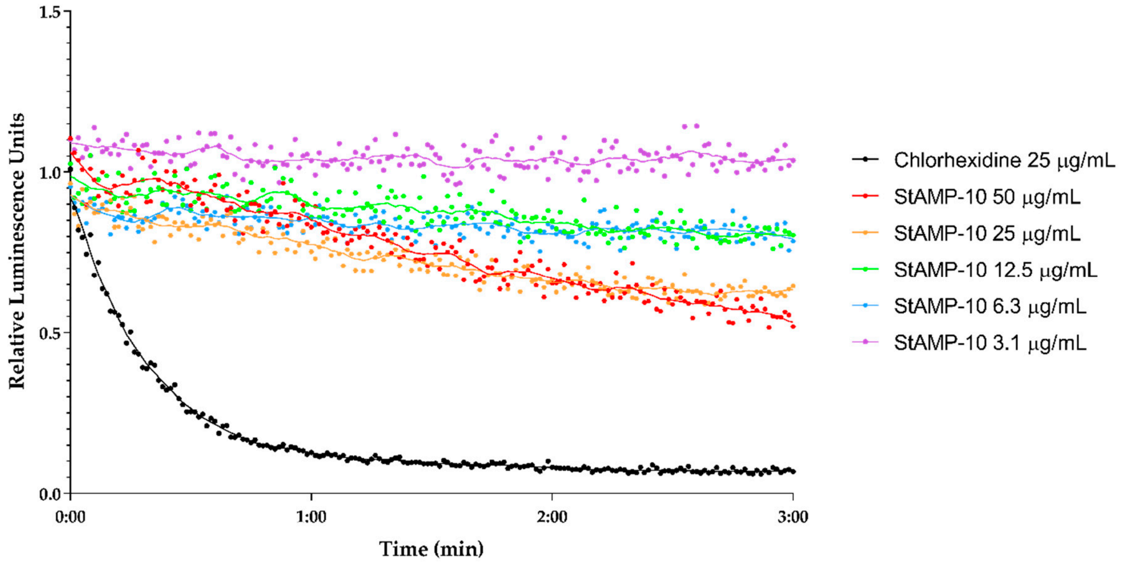

Figure S12. Kinetic of the antimicrobial effect on viability as measured by relative luminescence in *E. coli* (pCGLS-11) treated with chlorhexidine and different concentration of **StAMP-10**.

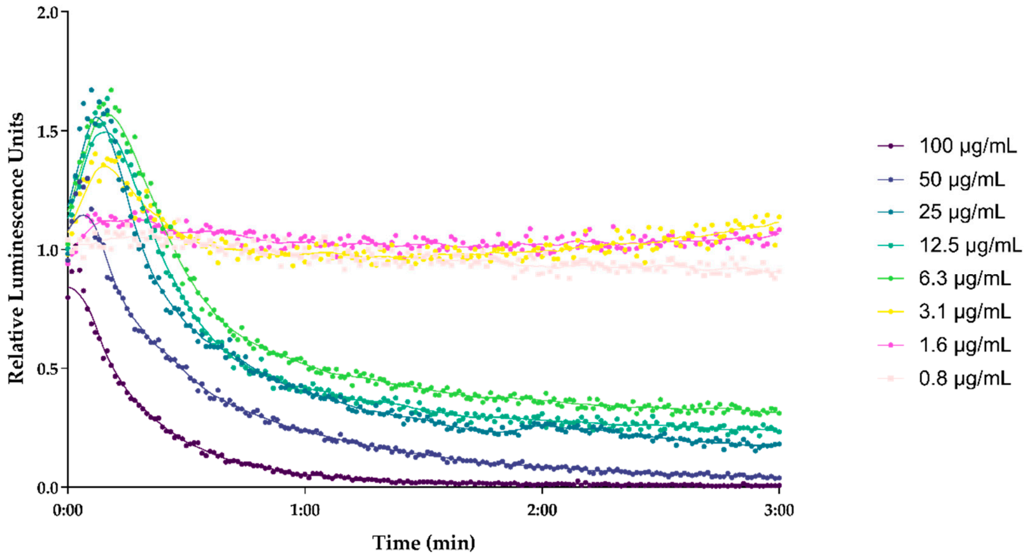

Figure S13. Kinetic of the antimicrobial effect on membrane integrity as measured by relative luminescence in *B. subtilis* (pCSS962) treated with different concentration of chlorhexidine.

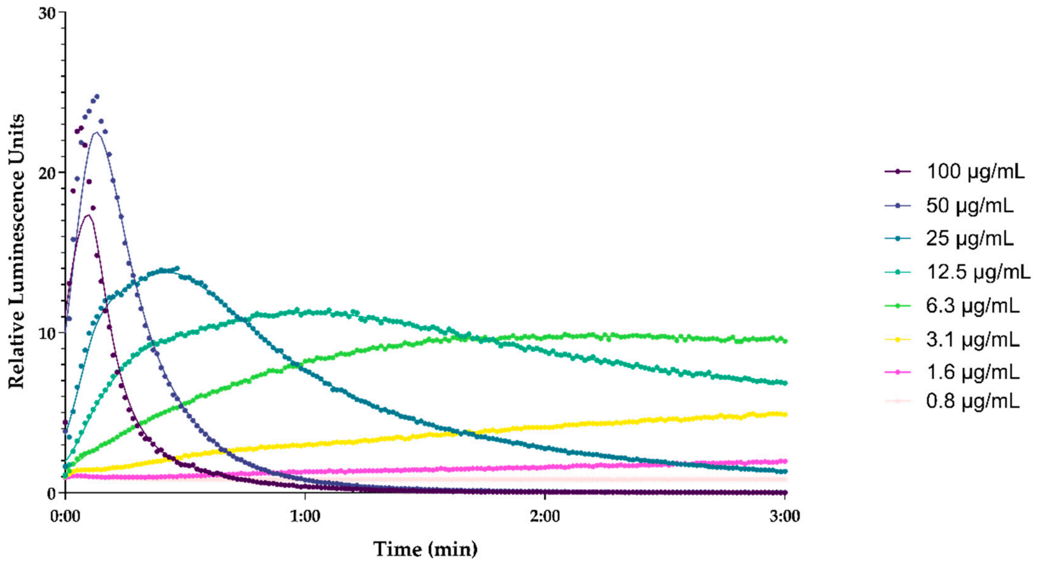

Figure S14. Kinetic of the antimicrobial effect on membrane integrity as measured by relative luminescence in *E. coli* (pCSS962) treated with different concentration of chlorhexidine.

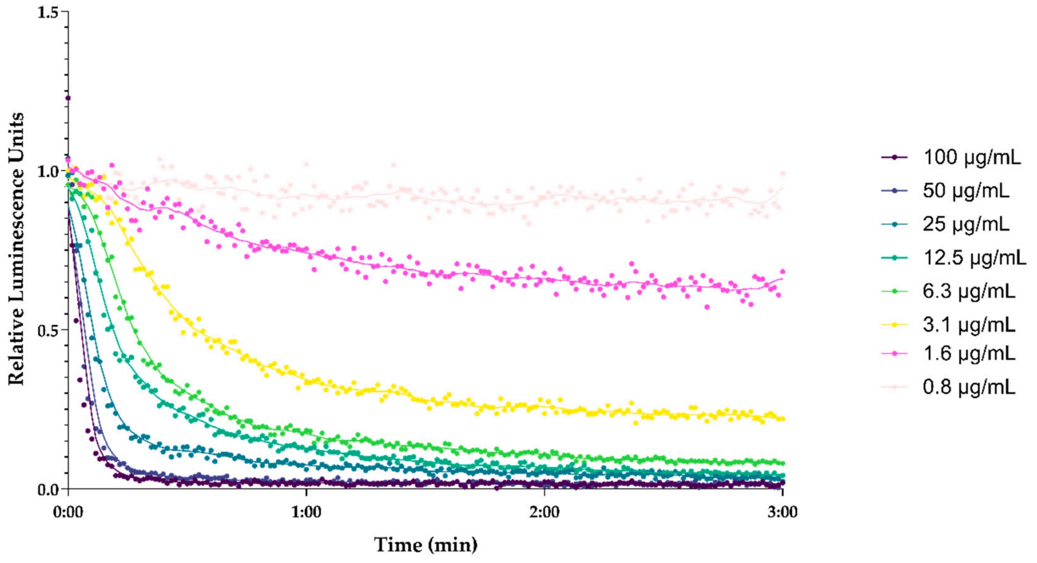

Figure S15. Kinetic of the antimicrobial effect on viability as measured by relative luminescence in *B. subtilis* (pCGLS-11) treated different concentration of chlorhexidine.

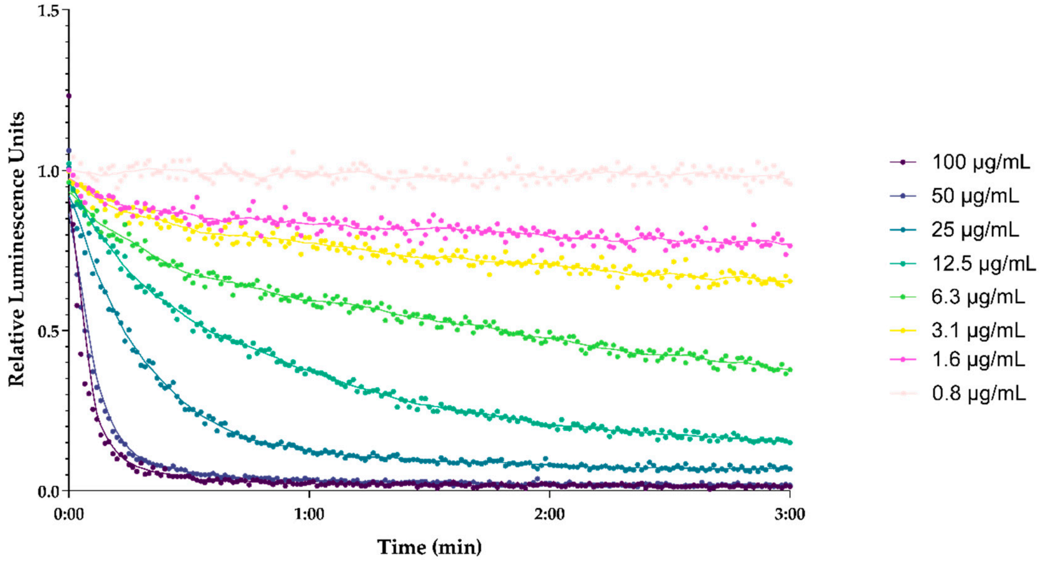

Figure S16. Kinetic of the antimicrobial effect on viability as measured by relative luminescence in *E. coli* (pCGLS-11) treated different concentration of chlorhexidine.

Table S1. Antimicrobial activity prediction of the designed StAMPs. SVM: support vector machines; RF: random forests; ANN: artificial neural networks; and DA: discriminant analysis.

Peptide	Sequence	CAMP$_{R3}$				ADAM
		SVM	RF	ANN	DA	SVM
StAMP-1	GKKPGGWKAK	0.968	0.559	AMP	0.884	2.85
StAMP-2	GKK**W**GGWKAK	0.998	0.533	AMP	0.887	3.23
StAMP-3	GKKP**W**GWKAK	0.999	0.625	AMP	0.979	2.85
StAMP-4	GKKPG**W**WKAK	0.997	0.623	AMP	0.979	2.85
StAMP-5	GKK**WW**GWKAK	1.000	0.605	AMP	0.980	3.14
StAMP-6	GKK**W**G**W**WKAK	1.000	0.605	AMP	0.980	3.14
StAMP-7	GKKP**WW**WKAK	1.000	0.724	AMP	0.997	2.79
StAMP-8	GKK**WWW**WKAK	1.000	0.830	AMP	0.998	2.90
StAMP-9	G**RR**P**WW**W**R**A**R**	0.999	0.634	AMP	0.993	1.36
StAMP-10	G**RRWWW**W**R**A**R**	1.000	0.649	AMP	0.995	1.97
StAMP-11	G**RR**P**LLLR**A**R**	0.918	0.583	AMP	0.907	1.82

Table S2. Molecular weight and purity of the StAMPs.

Peptide	Molecular weight (g/mol)	Purity (%)
StAMP-1	1055.28	97
StAMP-2	1144.37	98
StAMP-3	1184.44	98
StAMP-4	1184.44	98
StAMP-5	1273.53	95
StAMP-6	1273.53	98
StAMP-7	1313.59	99
StAMP-8	1402.69	100
StAMP-9	1425.65	100
StAMP-10	1514.74	100
StAMP-11	1206.49	97
